# Ammonium Accumulation and Cell Death in a Rat 3D Brain Cell Model of Glutaric Aciduria Type I

**DOI:** 10.1371/journal.pone.0053735

**Published:** 2013-01-10

**Authors:** Paris Jafari, Olivier Braissant, Petra Zavadakova, Hugues Henry, Luisa Bonafé, Diana Ballhausen

**Affiliations:** 1 Inborn Errors of Metabolism, Molecular Pediatrics, Lausanne University Hospital, Lausanne, Switzerland; 2 Inborn Errors of Metabolism, Biomedicine, Lausanne University Hospital, Lausanne, Switzerland; University of Edinburgh, United Kingdom

## Abstract

Glutaric aciduria type I (glutaryl-CoA dehydrogenase deficiency) is an inborn error of metabolism that usually manifests in infancy by an acute encephalopathic crisis and often results in permanent motor handicap. Biochemical hallmarks of this disease are elevated levels of glutarate and 3-hydroxyglutarate in blood and urine. The neuropathology of this disease is still poorly understood, as low lysine diet and carnitine supplementation do not always prevent brain damage, even in early-treated patients. We used a 3D *in vitro* model of rat organotypic brain cell cultures in aggregates to mimic glutaric aciduria type I by repeated administration of 1 mM glutarate or 3-hydroxyglutarate at two time points representing different developmental stages. Both metabolites were deleterious for the developing brain cells, with 3-hydroxyglutarate being the most toxic metabolite in our model. Astrocytes were the cells most strongly affected by metabolite exposure. In culture medium, we observed an up to 11-fold increase of ammonium in the culture medium with a concomitant decrease of glutamine. We further observed an increase in lactate and a concomitant decrease in glucose. Exposure to 3-hydroxyglutarate led to a significantly increased cell death rate. Thus, we propose a three step model for brain damage in glutaric aciduria type I: (i) 3-OHGA causes the death of astrocytes, (ii) deficiency of the astrocytic enzyme glutamine synthetase leads to intracerebral ammonium accumulation, and (iii) high ammonium triggers secondary death of other brain cells. These unexpected findings need to be further investigated and verified *in vivo.* They suggest that intracerebral ammonium accumulation might be an important target for the development of more effective treatment strategies to prevent brain damage in patients with glutaric aciduria type I.

## Introduction

Glutaric aciduria type I (GA-I; MIM #231670) is an autosomal recessive disorder caused by mutations in the mitochondrial enzyme glutaryl-CoA dehydrogenase (GCDH; EC 1.3.99.7; MIM *608801) [1]. Deficient GCDH activity affects tryptophan, lysine and hydroxylysine catabolism and results in the accumulation of glutaric acid (GA), 3-hydroxyglutaric acid (3-OHGA) and to a lesser extent glutaconic acid and glutaryl carnitine in body fluids and tissues [2], [3], [4]. GA-I belongs to a disease group in which in spite of generalized metabolic derangement, the symptoms are almost exclusively neurologic – the so-called “cerebral organic acidurias” [5]. After a pre-symptomatic period in which macrocephaly and/or subtle neurological signs can be noticed, most children with GA-I present with an acute encephalopathic crisis that usually occurs between the 6^th^ and 18^th^ month of life following an intercurrent infection or immunization. During this crisis, previously acquired motor skills are lost and permanent motor handicap remains. Neuroimaging shows bilateral destruction of caudate and putamen. A minority of patients experiences the insidious onset of this disease with delayed motor development and progressive dystonic cerebral palsy [1], [6], [7]. Early diagnosis and treatment allows preventing brain damage at least in part. Low lysine diet in combination with carnitine and anti-catabolic emergency treatment is the standard management in pre-symptomatic infants. However, up to one third of pre-symptomatically diagnosed patients (e.g. via newborn screening) encounter striatal injury or show fine motor and speech deficits [8], [9], [10], [11].

Nearly three decades of scientific research on the origin of neurological damage in organic acidurias have only partially uncovered the mechanisms of neurotoxicity in these disorders. The “toxic metabolite” and “trapping” hypotheses suggest that the production and accumulation of putative toxic metabolites in brain are involved in the pathomechanisms of organic acidurias. Following the “toxic metabolite” theory, GA and 3-OHGA are supposed to be putative brain cell toxins when produced and/or accumulated in the central nervous system of GA-I patients. These theories have directed the research towards neurotoxicity studies of the main metabolites accumulating in these diseases (reviewed in [9]). Three mechanisms have been suggested to be involved in the pathogenesis of this disease: i) excitotoxicity, ii) impairment of cerebral energy metabolism and iii) induction of oxidative stress [9].

A *Gcdh^−/−^* mouse model was generated that showed a biochemical phenotype similar to GA-I patients. Pathologically, these mice have a diffuse spongiform myelinopathy similar to that in human patients [12]. However, they do not present typical encephalopathic crises unless under high protein or high lysine diet. High protein diet is rapidly lethal, while 4 week-old *Gcdh^−/−^* mice under high lysine develop vasogenic edema, blood-brain barrier breakdown, neuronal loss, hemorrhage, paralysis and seizures, and die after 3–12 days. In contrast, most 8-week-old *Gcdh* ^−/−^ mice survive on high lysine diet, but develop white matter lesions, reactive astrocytes and neuronal loss [13]. Despite existing evidence for the role of GA and 3-OHGA in the neurotoxicity of GA-I, the neuropathogenesis of this disease still remains poorly understood.

We developed an *in vitro* model for the study of neurotoxicity in GA-I by exposing 3D organotypic rat brain cell cultures in aggregates to GA and 3-OHGA. This model mimics the production and accumulation of these metabolites during a metabolic crisis. We analyzed the effect of GA and 3-OHGA on brain cells in immature and more developed stages of the cultures. Cell morphology, cell death, and the metabolic profile in the culture medium have been studied.

## Materials and Methods

### Ethics Statement

This study was carried out in strict accordance to the Ethical Principles and Guidelines for Scientific Experiments on Animals of the Swiss Academy for Medical Sciences. The protocol was approved by the Ethics Committee for Animal Experimentation (Service de la consommation et des affaires vétérinaires, Epalinges, Switzerland; No. 1172.5). Sufficient amount of food and water for transportation and period before sacrificing of the rats was added by the commercial provider. All animals were sacrificed 48 hours after commercial delivery by decapitation with the use of a guillotine to avoid animal suffering.

### Rat 3D Organotypic Brain Cell Cultures in Aggregates

Pregnant Sprague-Dawley rats (Harlan; Netherlands) were sacrificed on day 15 of gestation. Fetal whole brains were extracted, pooled and mechanically dissociated. 3.6×10^7^ cells were grown in 8 ml of a serum-free, chemically-defined medium with 25 mM glucose and maintained under constant gyratory agitation at 37°C, in an atmosphere of 10% CO_2_ and 90% humidified air to form reaggregated 3D primary brain cell cultures as previously described [14], [15]. Media were replenished every three days from day-in-vitro (DIV) 5, by exchanging 5 ml of medium per culture. On the day of harvest aggregate pellets were washed three times with ice-cold PBS and either embedded for histology in cryoform (Tissue-Tek O.C.T. Compound, Sakura Finetek, Netherlands) and frozen in liquid nitrogen-cooled 2-methylbutane (Sigma-Aldrich, Germany); or directly frozen in liquid nitrogen and kept at −80°C until analysis.

### Treatment Protocol

**Figure 1:**
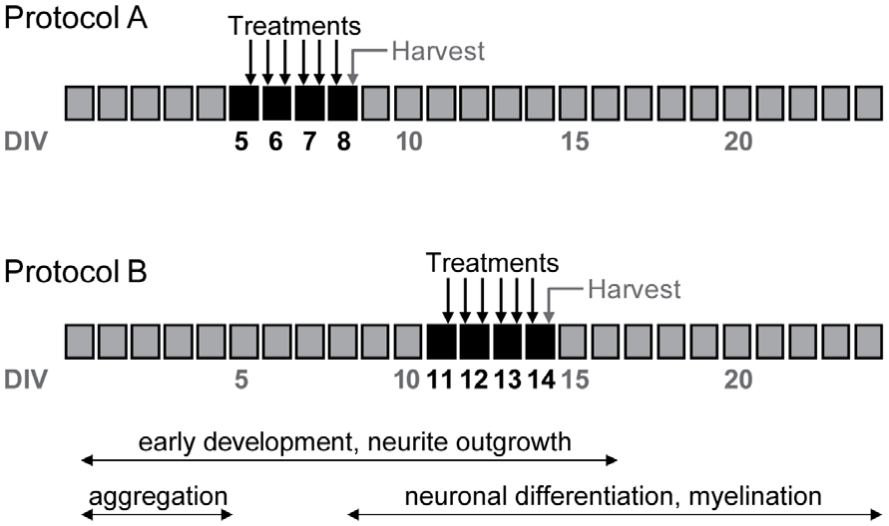
Treatment protocols. Cultures of aggregates were exposed to 1 mM GA and 3-OHGA at two time points representing different developmental stages of brain cell maturation (Protocols A and B). Metabolites were added 6 times every 12 hours (indicated by arrows) starting on DIV 5 in protocol A and on DIV 11 in protocol B (treatment days are indicated by black boxes) 12 hours after the change of the medium. Aggregates were harvested 5 hours after the last treatment at DIV 8 in protocol A and at DIV 14 in protocol B.

Cultures were treated with 1 mM glutaric acid (GA; Sigma-Aldrich, Germany) or 3-hydroxyglutaric acid (3-OHGA; Ernesto Brunet-Romero, Madrid, Spain) buffered in 25 mM HEPES with pH adjusted to 7.5. Cultures were exposed to one of the two metabolites 6 times every 12 hours at two different developmental stages starting from DIV 5 in protocol A or from DIV 11 in protocol B (**Figure 1**). Aggregates were harvested 5 hours after the last treatment at DIV 8 in protocol A and at DIV 14 in protocol B.

### Immunohistochemistry

Immunohistochemical staining was carried out on 16 µm aggregate cryosections using antibodies against different markers of brain cell types: phosphorylated medium weight neurofilament (p-NFM; clone NN18, Sigma-Aldrich, USA) for neurons [16], glial fibrillary acidic protein (GFAP; Millipore, USA) for astrocytes, galactocerebroside (GalC; Millipore, USA) on DIV 8 and myelin basic protein (MBP; Santa Cruz Biotechnology, USA) on DIV 14 for oligodendrocytes, and peroxidase-labeled isolectin B_4_ (Sigma-Aldrich, USA) on DIV 8 for microglia. Briefly, sections were fixed for 1 h in 4% paraformaldehyde in PBS at room temperature. Endogenous peroxidase activity was quenched with 1.5% H_2_O_2_ in PBS (Sigma-Aldrich, Germany) and non-specific antibody binding sites were blocked with 1% bovine serum albumin (Sigma-Aldrich, Germany) in PBS for 1 h. Primary antibodies diluted 1∶100 in 1% bovine serum albumin in PBS where applied to sections and further detected with anti-mouse or anti-rabbit IgG coupled to horseradish peroxidase (HRP, Bio-Rad Laboratories, USA). Staining was processed using the AEC Substrate Set for BD™ ELISPOT according to the manufacturer's protocol (BD Biosciences, USA). For negative controls, the primary antibodies were omitted resulting in no staining. The stained sections were mounted under FluorSave™ Reagent (Calbiochem, USA), observed and digitized using an Olympus BX50 microscope equipped with a UC30 digital camera (Olympus, Japan).

### Immunofluorescence

Detection of cleaved caspase-3 in aggregates was performed with the Tyramide Signal Amplification Kit (Life Technologies, USA). Aggregate cryosections (16 µm) were subjected to the same procedure as described above for immunohistochemistry. Non-specific antibody binding sites were blocked for 1 h at room temperature with the blocking buffer of the kit. The primary antibody against the large fragment (17/19 kDa) of activated caspase-3 (Cell Signaling Technology, USA), diluted 1∶1000 in blocking buffer, was applied to sections overnight at 4°C. After washing, sections were incubated with a HRP anti-rabbit IgG secondary antibody (provided by the kit) for 1 h. Peroxidase staining was performed using Alexa Fluor® 555-labeled tyramide diluted 1∶200 in amplification buffer (provided by the kit) and applied to sections for 10 min. Negative controls were processed the same but omitting the primary antibody resulting in no staining. Sections were mounted under FluorSave™ reagent. The sections were observed and photographed with an Olympus BX50 microscope equipped with a UC30 digital camera.

### In situ Cell Death Detection

To detect typical features of apoptosis (fragmented nuclei, apoptotic bodies), nuclear DNA was stained using the blue fluorescent 4',6-diamidino-2-phenylindole (DAPI, Invitrogen, USA). Aggregate cryosections (16 µm) were incubated with DAPI for 10 min at room temperature. *In situ* detection of cell death was performed using terminal deoxynucleotidyl transferase (TdT)-mediated dUTP nick end labeling (TUNEL) on 16 µm cryosections of aggregates. TUNEL staining was performed according to supplier recommendations using the *In Situ* Cell Death Detection Kit with Fluorescein (Roche Applied Science, Switzerland) resulting in green fluorescence in dying cells.

### Western Blot Analysis

Aggregates were homogenized in 150 mM sodium chloride, 50 mM Tris-HCl, pH 8, 1% NP-40 (Sigma-Aldrich, Germany) and Protease Inhibitor Cocktail - Complete Mini (Roche Applied Science, Switzerland) and sonicated for 5 seconds. Lysates were cleared by centrifugation at 12′000 rpm for 30 min at 4°C. After dilution, protein content was measured by bicinchoninic acid assay (Thermo Scientific, USA) and diluted with NuPAGE® LDS Sample Buffer (Life Technologies, USA) to a final concentration of 1.2 µg/µl. Samples were heated at 70°C for 10 min and resolved on NuPAGE® 4–12% Bis–Tris Gel (for p-NFM) or NuPAGE® 12% Bis–Tris Gel (for GFAP, MBP, actin and caspase-3) using NuPAGE® MOPS SDS Running Buffer (Life Technologies, USA) at a constant voltage (200 V, 60 min). Proteins were transferred onto Immobilon-FL PVDF, 0.45 µm membranes (Millipore, USA). Membranes were blocked with 5% non-fat dry milk in TBS-Tween (20 mM Trizma base, 137 mM NaCl, 0.05% Tween, pH 7.6) for 1 h at room temperature. After blocking, the membranes were incubated overnight with different primary antibodies against GFAP, MBP, p-NFM, Actin (I-19) (Santa Cruz Biotechnology, USA) or full-length (35 kDa) and large fragment (17/19 kDa) of caspase-3 (Cell Signaling Technology, USA) diluted 1∶1000 in 3% dry milk and TBS-Tween. The membranes were probed with HRP-conjugated goat anti-mouse IgG or goat anti-rabbit IgG (1∶3000; Vector laboratories, USA) and developed by chemiluminescence (ECL Western Blotting Detection Reagents; GE Healthcare, France). Blots were stripped (ReBlot Plus Mild Antibody Stripping Solution; Millipore, USA) and re-probed with antibody against actin as the loading control. Images were taken with a Luminescent Image Analyzer LAS-4000 (Fujifilm; Life Science) and quantified with the public Java-based image processing program ImageJ (National Institutes of Health). Data were acquired in arbitrary densitometric units and transformed to percentages of the densitometric levels obtained from scans of control samples visualized on the same blots.

### Measure of Basic Metabolites and Amino Acids in the Culture Media

Ammonium was measured on an Integra automatic analyzer (Roche); glucose, lactate and lactate dehydrogenase (LDH) were measured on a Modular automatic analyzer (Roche); free amino acids were analyzed on a Beckman 6300 amino acid analyzer; as described previously [17]; lactate and pyruvate for measurement of the lactate/pyruvate ratio were measured by GC/MS (HP 6890 N, Agilent Technologies).

### Expression of GCDH in Brain Cells

Non-radioactive *in situ* hybridization for *Gcdh* mRNA, making use of digoxygenin-labeled riboprobes transcribed from *Gcdh* cDNA, was performed on adult rat brain cryosections (16 µm), as described [14]. Sections were then co-labeled by immunohistochemistry with specific antibodies for neurons (neuronal nuclei protein; NeuN; Millipore, USA), astrocytes (GFAP) and oligodendrocytes (MBP).

### Statistics

All data points are expressed as mean ± standard deviation (SD). Statistical difference was determined with Student’s *t*-test.

## Results

### GA and 3-OHGA Exposure Altered the Morphology of Developing Brain Cells

#### Neurons

**Figure 2:**
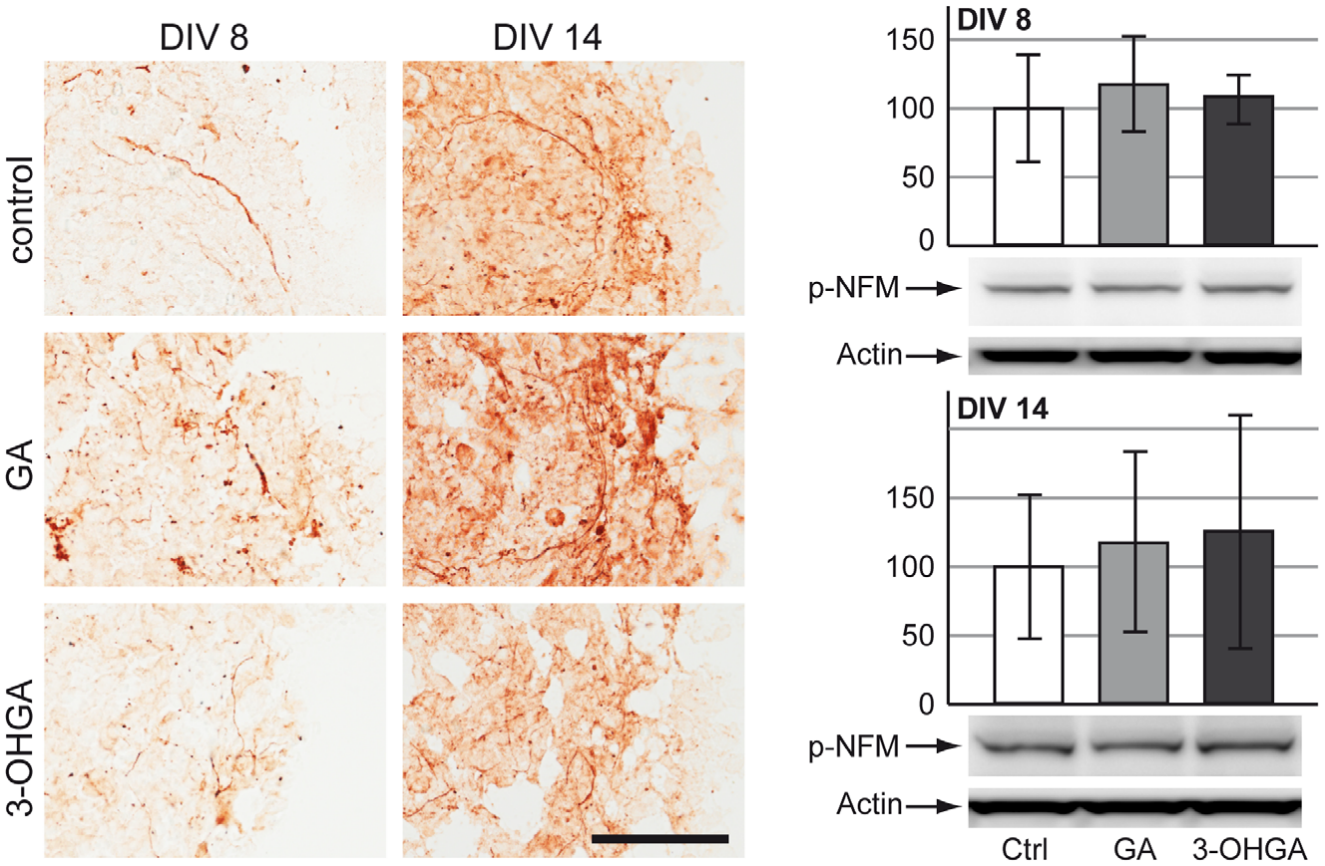
Effects of GA and 3-OHGA on neurons. (Left panel) Immunohistochemical staining for phosphorylated medium weight neurofilament (p-NFM) on cryosections of cultures derived from protocol A (DIV 8) and protocol B (DIV 14). Scale bar: 100 µm. (Right panel) Representative western blots with data quantification of whole-cell lysates for p-NFM for protocol A (DIV 8, above) and protocol B (DIV 14, below). Actin was used as a loading control. The quantifications of p-NFM are expressed as percentage of respective controls. The values represent the mean ± SD from 3 replicates taken from 2 independent experiments.

Immunohistochemical staining for p-NFM on cryosections from cultures exposed to GA and 3-OHGA did not show any eminent effects on the expression and distribution of p-NFM in both treatment protocols. We observed normal axonal elongation and no aberrant accumulation of this protein in the cellular body. However, we could see a substantial overall tissue damage in the aggregates exposed to 3-OHGA under protocol A (DIV 8) and even more remarkably under protocol B (DIV 14) (**Figure 2, left panel**). Quantitative analysis of p-NFM expression by western blotting did not reveal any significant changes in the treated cultures compared to control (**Figure 2, right panel**).

#### Astrocytes

**Figure 3:**
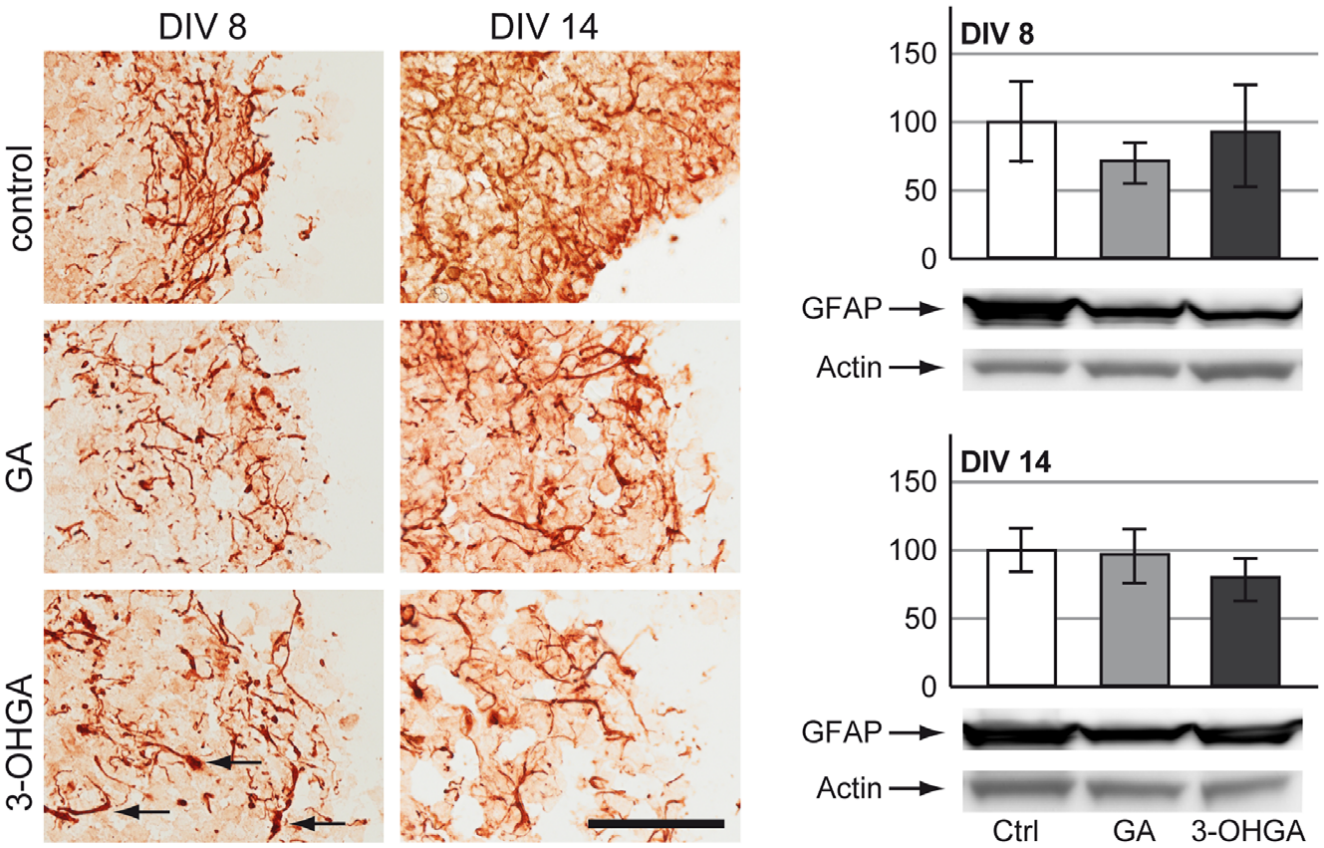
Effects of GA and 3-OHGA on astrocytes. (Left panel) Immunohistochemical staining for glial fibrillary acidic protein (GFAP) on cryosections of cultures derived from protocol A (DIV 8) and protocol B (DIV 14). Swollen proximal fibers are indicated by black arrows. Scale bar: 100 µm. (Right panel) Representative western blots with data quantification of whole-cell lysates for GFAP for protocol A (DIV 8, above) and protocol B (DIV 14, below). Actin was used as a loading control. The quantifications of GFAP levels are expressed as percentage of respective controls. The values represent the mean ± SD from 3 replicates taken from 2 independent experiments.

GFAP immunohistochemical staining revealed a decrease in astrocytic fiber density in cultures exposed to 3-OHGA and to a lesser extent also in those treated with GA, for both treatment protocols (**Figure 3, left panel**). Interestingly, 3-OHGA exposure led to appearance of proximal fiber swelling in surviving astrocytes in immature cultures (DIV 8) as indicated by arrows in **Figure 3**. Similarly to the observations for the p-NFM signal in neurons, we could again see severe tissue damage in 3-OHGA-exposed aggregates in protocol B (DIV 14), suggesting massive cell death. GFAP expression in surviving astrocytes was not significantly altered as shown by western blot analyses (**Figure 3, right panel**).

#### Oligodendrocytes

**Figure 4:**
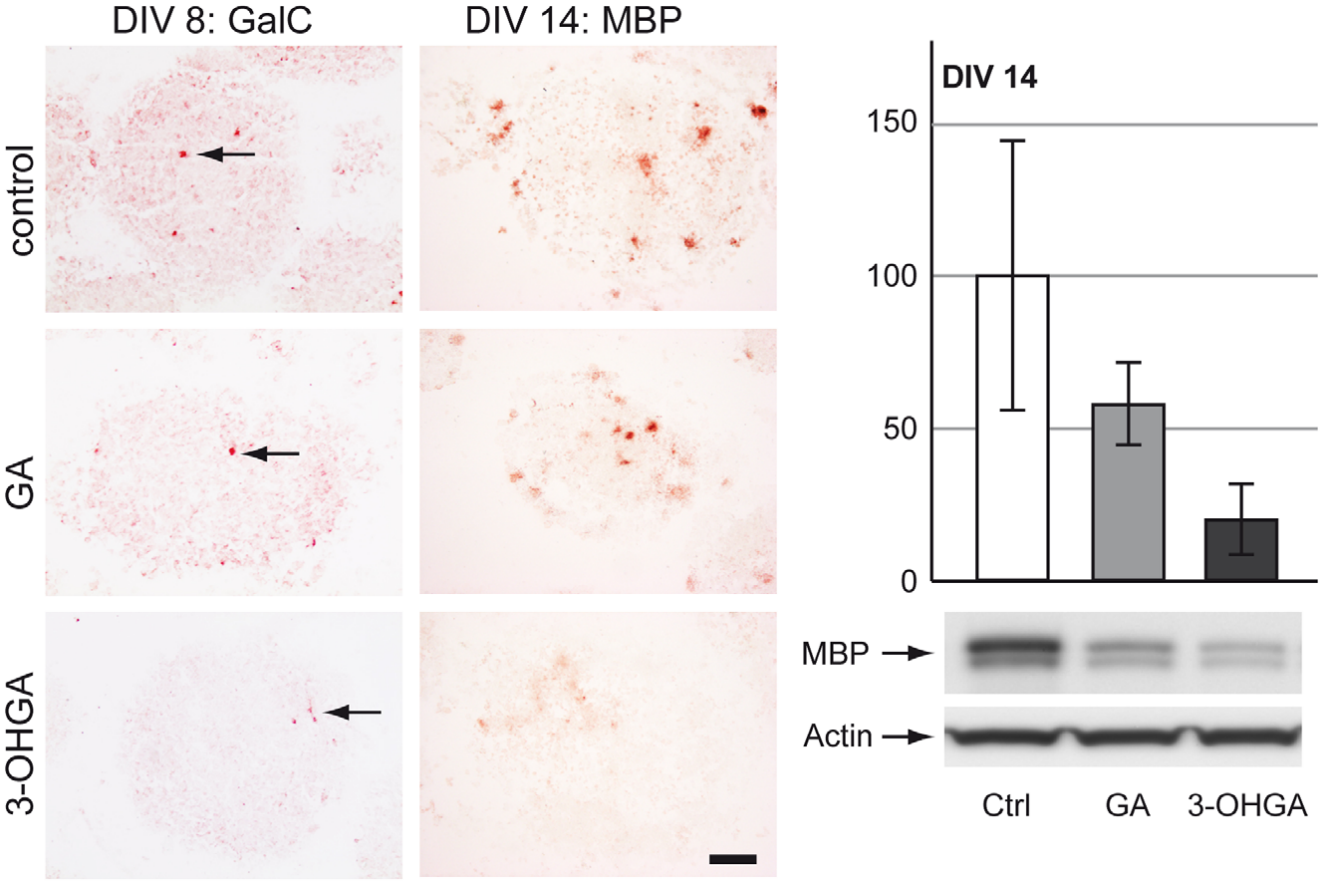
Effects of GA and 3-OHGA on oligodendrocytes. (Left panel) Immunohistochemical staining for galactocerebroside (GalC, DIV 8) and myelin basic protein (MBP, DIV 14) on cryosections derived from protocol A (DIV 8) and protocol B (DIV 14). Oligodendrocytes are indicated by black arrows. Scale bar: 100 µm. (Right panel) Representative western blots with data quantification of whole-cell lysates for MBP for protocol B (DIV 14). Actin was used as a loading control. The quantifications of MBP levels are expressed as percentage of respective controls. The values represent the mean ± SD from 3 replicates taken from 2 independent experiments.

3-OHGA-exposure, and to a lesser extent GA-exposure, resulted in a substantial decrease of MBP staining under protocol B (DIV 14) (**Figure 4, left panel**). Western blot analysis confirmed decreased MBP expression under the same conditions (**Figure 4, right panel**). We could not see any effect for MBP staining in protocol A since the expression of MBP protein is very low in the immature developmental stages (data not shown). In order to discriminate whether the observed signal loss is a result of oligodendrocytic death or altered differentiation and/or myelination, we performed immunohistochemical staining for GalC, one of the earliest markers of oligodendrocytes. Only slight reduction of GalC signal was observed in the cultures treated with 3-OHGA on DIV 8 (**Figure 4, left panel**) and no difference was seen with any of the two metabolites on DIV 14 (data not shown).

#### Microglia

The presence of microglia was tested by immunostaining for isolectin B_4_ at DIV 8. No interesting changes were observed (data not shown).

### Biochemical Parameters in Culture Media after Exposure to GA and 3-OHGA

#### Glucose and Lactate

**Figure 5:**
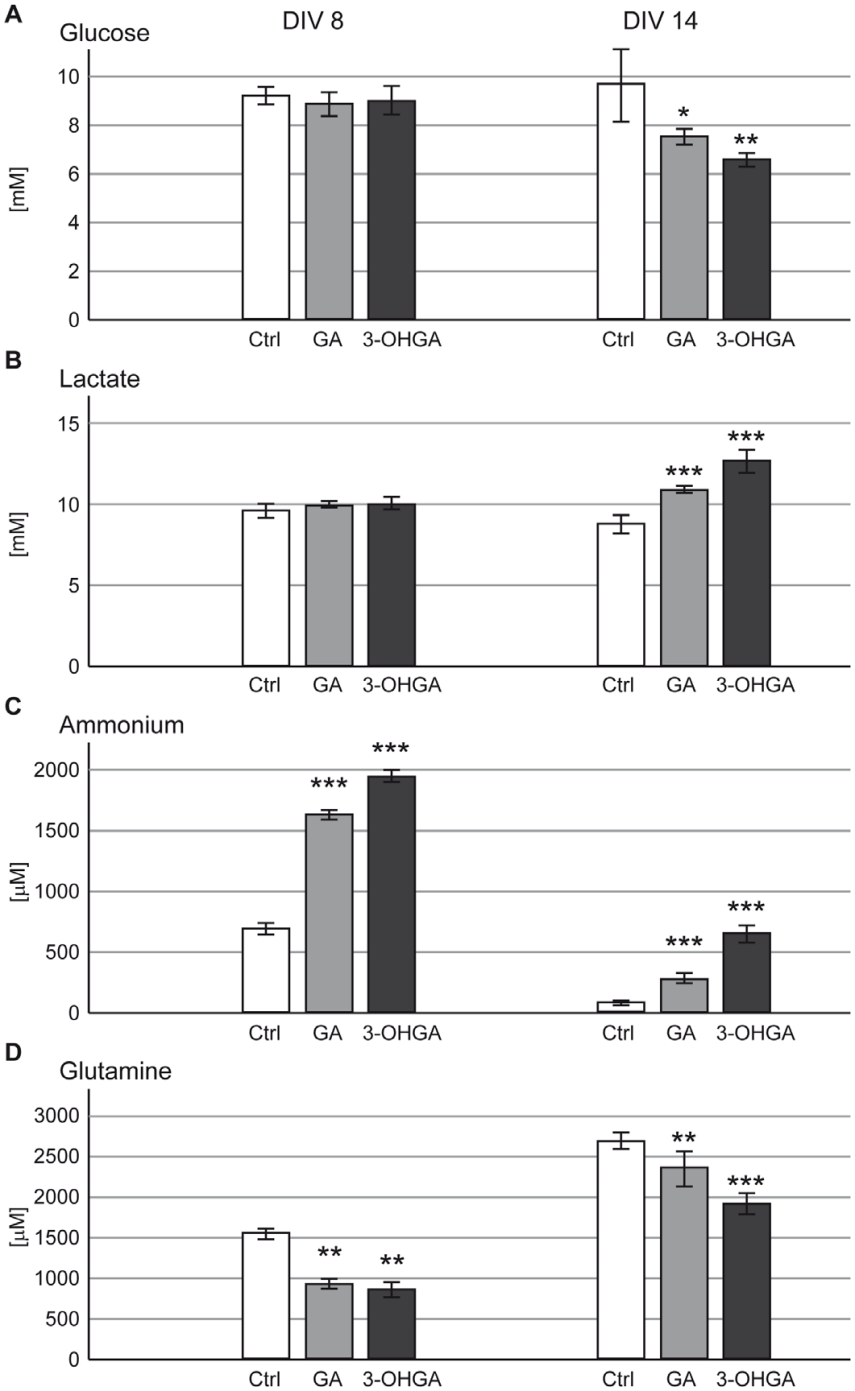
Effects of GA and 3-OHGA on biochemical parameters measured in culture medium. Glucose (A), lactate (B), ammonium (C) and glutamine (D) were measured in the medium of cultures treated with protocols A (DIV 8) or B (DIV 14). Mean ± SD of 7 replicate cultures assessed by Student’s *t*-test; *p<0.05, **p<0.01, *** p<0.001.

As compared to controls, GA and 3-OHGA exposure caused a significant decrease in the glucose levels under protocol B (DIV 14), while the glucose levels of immature cultures (protocol A, DIV 8) were not significantly changed (**Figure 5A**). In parallel, a significant increase in lactate levels was observed in the medium of treated DIV 14 cultures. Lactate release into medium remained unchanged in immature DIV 8 cultures (**Figure 5B**). The lactate/pyruvate ratio was increased in the medium of DIV 14 3-OHGA-exposed cultures (55.4 under 1 mM 3-OHGA *versus* 26.2 in controls; mean of duplicates for each condition).

#### Ammonium and Glutamine

A massive increase in ammonium concentrations was measured in the culture media after exposure to 3-OHGA and GA under both protocols (DIV 8 and 14) (**Figure 5C**). Among amino acids measured in the culture medium, a significant decrease was observed on glutamine levels in all cultures exposed to GA and 3-OHGA under both protocols (**Figure 5D**).

### Increased Cell Death in Developing Brain Cells After Exposure to GA and 3-OHGA

**Figure 6:**
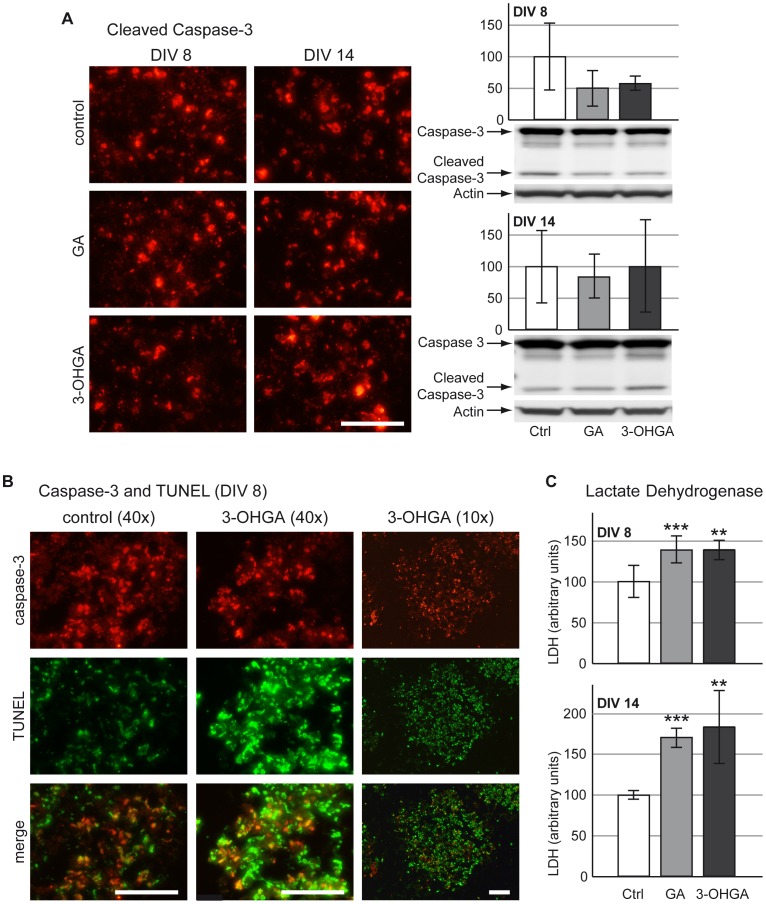
Evaluation of cell death after treatment with GA and 3-OHGA. (A; left panel) Immunohistochemical staining for cleaved caspase-3 (red signal). Scale bar: 100 µm. (A; right panel) Representative western blots with data quantification of whole-cell lysates for full length caspase-3 and the large fragment of cleaved (e.g. activated) caspase-3 for protocol A (DIV 8, above) and protocol B (DIV 14, below). Actin was used as a loading control. The quantifications of cleaved caspase-3 are expressed as percentage of respective controls. The values represent the mean ± SEM from 3 replicates taken from 2 independent experiments. (B) *In situ* cell death assay with TUNEL (green signal) and cleaved caspase-3 (red signal) on DIV 8 (protocol A). Merge of both signals leads to double-stained cells appearing in yellow. Scale bar: 100 µm. (C) LDH in culture medium of cultures from protocol A (DIV 8, above) and protocol B (DIV 14, below). Mean ± SD of 7 replicate cultures assessed by Student’s *t*-test; **p<0.01, *** p<0.001.

Lactate dehydrogenase (LDH) was measured in culture medium and was significantly increased after 3-OHGA- and GA-exposure in both protocols (DIV 8 and DIV 14) (**Figure 6C**). This observation indicated an increase of cell death in these cultures. To evaluate cell death, we performed TUNEL, DAPI and activated caspase-3 immunofluorescence staining. DAPI staining did not show an increased appearance of nuclear fragmentation and apoptotic bodies in cultures under both protocols, as compared to control (data not shown). Immunofluorescence staining for cleaved caspase-3 revealed no difference in the number of positively stained cells (e.g. apoptotic) in cultures exposed to GA and 3-OHGA in both treatment protocols as compared to control (**Figure 6A, left panel**). Accordingly, no significant changes were observed on activated caspase-3 level assessed by western blotting under both metabolites treatment at DIV 14. In protocol A, a tendency of activated caspase-3 to decrease in GA- and 3-OHGA-exposed aggregates was observed, however, the changes were not significant (**Figure 6A, right panel**). Interestingly, TUNEL labeling (green staining) showed an important signal increase for cultures treated with 3-OHGA on DIV 8, which only partially co-localized (yellow staining) with cleaved caspase-3-labeled apoptotic cells (red signal) (**Figure 6B**). This suggests an induction of non-apoptotic cell death in developing brain cells under 3-OHGA exposure. TUNEL signal in 3-OHGA exposed cultures was homogenously distributed over the entire aggregate, suggesting that 3-OHGA diffused well from the medium into the whole aggregate (**Figure 6B**, 10×magnifications).

### GCDH Expression in Adult Rat Brain

**Figure 7:**
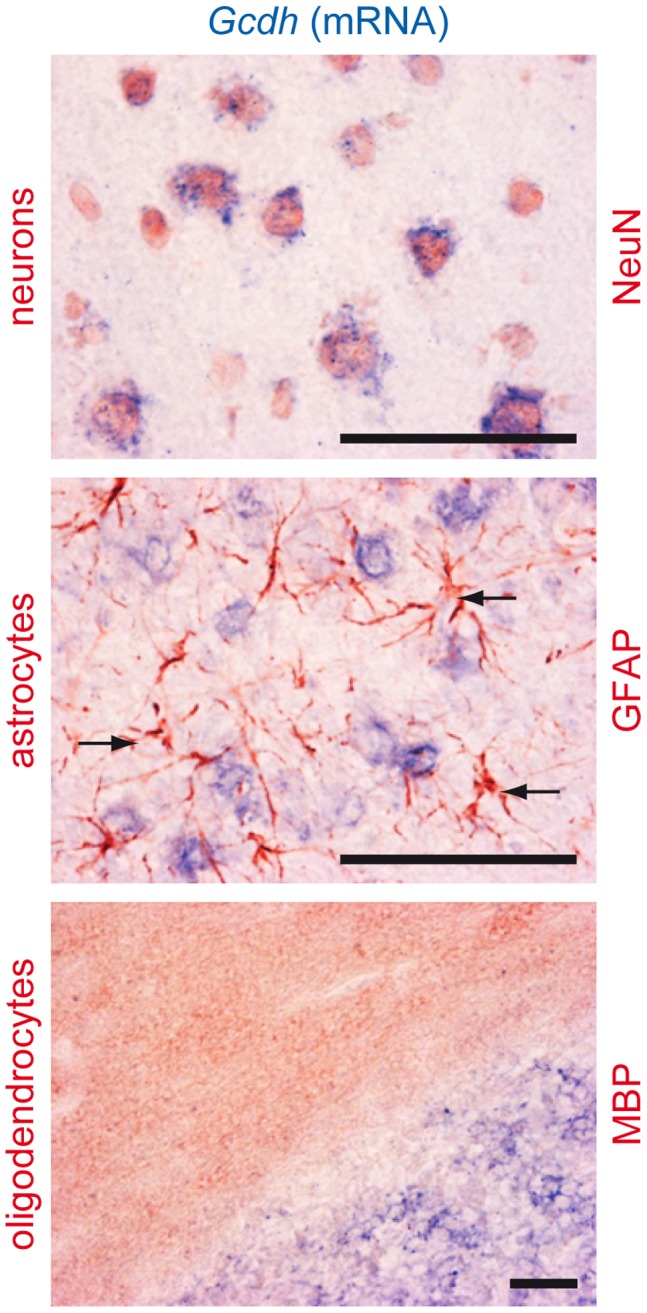
Expression of GCDH in neurons, astrocytes and oligodendrocytes. *In situ* hybridization for *GCDH* mRNA in adult rat brain (16 µm cryosections), co-labeled by immunohistochemistry for specific markers of neurons (NeuN), astrocytes (GFAP) or oligodendrocytes (MBP). Top and central panels show expression of *GCDH* mRNA (blue signal) in cortical neurons (top; NeuN, red signal), while *GCDH* mRNA could not be detected in cortical astrocytes (central; GFAP, red signal, arrows pointing at astrocytic cell bodies). Bottom panel shows *GCDH* mRNA (blue signal) in granular neurons of cerebellum, while *GCDH* mRNA appears absent from adjacent oligodendrocytes in white matter of cerebellum (bottom; MBP, red signal). Scale bar: 100 µm.

*Gcdh* mRNA was found expressed in the whole rat brain, exclusively in neurons (**Figure 7**). No *Gcdh* mRNA was detectable in astrocytes or in oligodendrocytes.

## Discussion

We used 3D organotypic brain cell cultures in aggregates to explore, *in vitro*, the effects of the two main metabolites (GA and 3-OHGA) accumulated in body fluids of subjects affected by GA-I. Our *in vitro* model is particularly suitable for studying neurotoxicity because the aggregates contain all types of brain cells with their spontaneous connections between each other. In addition, this model reproduces early phases of brain development and has proven to be optimal to study differential effects of metabolic derangements (such as hyperammonemia) in developing brain compared to adult brain tissue [14], [15], [16], [18]. This is particularly important when studying a disorder in which the most dramatic brain damage occurs in early childhood.

In our study, we could confirm that both GA and 3-OHGA were deleterious for brain cells during development, but 3-OHGA turned out to be the most toxic metabolite. The most striking effect of 3-OHGA on the aggregates was massive tissue destruction with wide areas of cell death, which was evident on DIV 14 (Figures 2 and 3). In both developmental stages (DIV 8 and 14) astrocytes appeared more susceptible to GA and 3-OHGA toxicity than neurons and oligodendrocytes, showing important morphological changes (decrease in fiber density and swelling of proximal fibers). These results are in line with the findings in the *Gcdh^−/−^* mouse which under high lysine diet show reactive astrocytes [13]. Surprisingly, neurons did not show any significant alteration in our model. This might be due to their origin from wild type animals, thus expressing *Gcdh*, or a too short observation time if they die secondarily to astrocytes and consecutive ammonium accumulation (see below). The observation of primary astrocytic reactivity and secondary neuronal death has already been described in a rat model using intracerebroventricular injection of GA [19]. Oligodendrocytes reacted differently. While under GA and 3-OHGA exposures GalC labeling was not significantly altered on both DIV 8 and DIV 14, MBP expression on DIV 14 was decreased. These results suggest delayed or altered differentiation of oligodendrocytes rather than their death. Delayed myelination and supratentorial white matter lesions have been described in GA-I patients with and without preceding encephalophatic crises [6] and in the *Gcdh^−/−^* mouse under high lysine diet [13].

Massive cell death in cultures exposed to both metabolites was also confirmed by LDH increase and by striking increase of *in situ* cell death detection by TUNEL assay. As treated cultures did not show an increase of activated caspase-3, we conclude that the GA- and 3-OHGA-induced cell death was mainly non-apoptotic. As astrocytes appeared the most affected cells (see above), and as 3-OHGA (and GA) exposure caused a massive elevation of ammonium in parallel with a decrease of glutamine in the culture medium (see above), this study suggests that astrocytes exposed to 3-OHGA (and GA) are the primary cells to die. Astrocyte susceptibility to 3-OHGA and GA toxicity may be due to the absence of *Gcdh* expression in these cells (see **Figure 7**).

Unexpectedly, a striking increase of ammonium was observed in the medium of cultures exposed to GA and 3-OHGA. The decreasing ammonium level of control cultures between DIV 8 and DIV 14 can be explained by the maturation of astrocytes, which increases their capacity to metabolize ammonium. Hyperammonemia can be seen occasionally in patients with organic acidurias during metabolic decompensation, but is usually moderate and transitory, and was so far thought to be of hepatic origin. In contrast, the observed ammonium accumulation in our *in vitro* model indicates that significant amounts of ammonium are produced by brain cells under the action of GA and 3-OHGA, suggesting a central liberation of ammonium in GA-I. Following the guidelines for GA-I, ammonium is not routinely determined during an acute illness [10], [11], but could be worth to be measured in CSF.

Ammonium is known to be toxic for brain cells causing reduced axonal elongation [16] as well as neuronal and oligodendrocytic cell death [15], [18], which correlates with the brain atrophy and white matter changes observed in patients with primary hyperammonemias [20]. Its detection in brain cell cultures challenged with GA and 3-OHGA immediately raises the question of a potential role for ammonium in brain damage occurring in GA-I patients. As urea cycle is not active in central nervous system, ammonium produced during amino acid catabolism is mainly detoxified through amination of glutamate to glutamine by the enzyme glutamine synthetase. This enzyme is exclusively expressed in astrocytes. In previous studies we have shown that ammonium concentrations up to 5 mM are not toxic for astrocytes, but induce cell death in neurons and oligodendrocytes [18]. Thus, we can conclude that the 3-OHGA-induced primary astrocytic death is not related to high ammonium levels, but might be secondarily followed by neuronal and oligodendrocytic death triggered by ammonium accumulation. This hypothesis is supported by the observation of neuronal loss in the *Gcdh^−/−^* mouse model [13].

Analysis of media from treated and control cultures on DIV 14 showed a marked increase in lactate with concomitant decrease in glucose concentrations. This combination can be observed in plasma of children with GA-I during acute encephalopathic crises. Underlying mechanisms may be the inhibition of the TCA cycle and/or respiratory chain with shift to lactate at the end of glycolysis, which is also supported by the 2-fold increase of the lactate/pyruvate ratio observed under 3-OHGA exposure. Lamp *et al.* have shown that 3-OHGA and GA inhibit astrocytic efflux and neuronal uptake of TCA cycle intermediates. These results suggest that elevated levels of 3-OHGA and GA may lead to neuronal injury and cell death via disruption of TCA cycle activity [21]. Direct effects on the respiratory chain have been reported controversially: While a recent report failed to prove changes on the activity of the different respiratory chain complexes in the *Gcdh^−/−^* mouse during metabolic crisis [22], other publications confirmed an impact of GA and/or 3-OHGA on the mitochondrial energy metabolism [23], [24]. TCA and respiratory chain dysfunction is also assumed by the “toxic metabolite” hypothesis that postulates an interference of these organic acids with mitochondrial energy metabolism [23]. This is consistent with the finding of mainly non-apoptotic (likely necrotic) cell death, which has been shown to be prevalent in animal models of mitochondrial dysfunction [25] as well as in neuropathology of humans with Leigh syndrome [26].

Our previous results on ammonium toxicity combined with the new results on our GA-I *in vitro* model suggest the following three-step model for brain damage in GA-I: (i) 3-OHGA (and GA) cause via a so far unknown mechanism massive cell death of astrocytes; (ii) loss of the astrocytic subpopulation results in deficiency of glutamine synthetase activity leading to ammonium accumulation; and (iii) ammonium accumulation results in secondary death of other brain cells (neurons and oligodendrocytes).

### Conclusions

In an *in vitro* brain cell culture model for GA-I, we confirm the toxicity of the two main accumulating metabolites, GA and 3-OHGA, on brain cells; the latter being the most deleterious substance. Our data allow the following conclusions: (i) 3-OHGA leads to massive cell death most likely of non-apoptotic origin; (ii) among the different cellular subpopulations in our model, astrocytes appeared to be the most vulnerable cells; (iii) ammonium accumulation might be secondary to the loss of the astrocytic enzyme glutamine synthetase and play a role in GA-I-related brain damage; (iv) indirect signs of impaired energy metabolism seem to support previous studies suggesting participation of this mechanism in the neuropathogenesis of GA-I. We suggest a three-step model for brain damage in GA-I. This model, if confirmed *in vivo*, may explain why investigation of direct neurotoxicity of GA and 3-OHGA has been difficult so far. It may open new therapeutic approaches for neuroprotection focused on the inhibition/detoxification of intracerebrally-produced ammonium. We might thus be one step closer to the prevention of the destructive processes that cause permanent handicap in GA-I.

## References

[1] Hedlund GL, Longo N, Pasquali M (2006) Glutaric acidemia type 1. American journal of medical genetics Part C, Seminars in medical genetics 142C: 86–94. 10.1002/ajmg.c.30088 PMC2556991 16602100

[2] Sauer SW, Okun JG, Fricker G, Mahringer A, Muller I, et al (2006) Intracerebral accumulation of glutaric and 3-hydroxyglutaric acids secondary to limited flux across the blood-brain barrier constitute a biochemical risk factor for neurodegeneration in glutaryl-CoA dehydrogenase deficiency. Journal of neurochemistry 97: 899–910. 16573641 10.1111/j.1471-4159.2006.03813.x

[3] Strauss KA, Morton DH (2003) Type I glutaric aciduria, part 2: a model of acute striatal necrosis. American journal of medical genetics Part C, Seminars in medical genetics 121C: 53–70. 10.1002/ajmg.c.20008 12888986

[4] Strauss KA, Puffenberger EG, Robinson DL, Morton DH (2003) Type I glutaric aciduria, part 1: natural history of 77 patients. American journal of medical genetics Part C, Seminars in medical genetics 121C: 38–52. 10.1002/ajmg.c.20007 12888985

[5] Hoffmann GF, Meier-Augenstein W, Stöckler S, Surtees R, Rating D, et al (1993) Physiology and pathophysiology of organic acids in cerebrospinal fluid. Journal of inherited metabolic disease 16: 648–669. 8412012 10.1007/BF00711898

[6] Harting I, Neumaier-Probst E, Seitz A, Maier EM, Assmann B, et al (2009) Dynamic changes of striatal and extrastriatal abnormalities in glutaric aciduria type I. Brain : a journal of neurology. 132: 1764–1782. 10.1093/brain/awp112 19433437

[7] Hoffmann GF, Zschocke J (1999) Glutaric aciduria type I: from clinical, biochemical and molecular diversity to successful therapy. Journal of inherited metabolic disease 22: 381–391. 10407775 10.1023/a:1005543904484

[8] Beauchamp MH, Boneh A, Anderson V (2009) Cognitive, behavioural and adaptive profiles of children with glutaric aciduria type I detected through newborn screening. Journal of inherited metabolic disease. 10.1007/s10545-009-1167-z 19466578

[9] Jafari P, Braissant O, Bonafe L, Ballhausen D (2011) The unsolved puzzle of neuropathogenesis in glutaric aciduria type I. Molecular genetics and metabolism. 104: 425–437. 10.1016/j.ymgme.2011.08.027 21944461

[10] Kölker S, Christensen E, Leonard JV, Greenberg CR, Boneh A, et al (2011) Diagnosis and management of glutaric aciduria type I–revised recommendations. Journal of inherited metabolic disease 34: 677–694. 21431622 10.1007/s10545-011-9289-5 PMC3109243

[11] Kölker S, Christensen E, Leonard JV, Greenberg CR, Burlina AB, et al (2007) Guideline for the diagnosis and management of glutaryl-CoA dehydrogenase deficiency (glutaric aciduria type I). Journal of inherited metabolic disease 30: 5–22. 17203377 10.1007/s10545-006-0451-4

[12] Koeller DM, Woontner M, Crnic LS, Kleinschmidt-DeMasters B, Stephens J, et al (2002) Biochemical, pathologic and behavioral analysis of a mouse model of glutaric acidemia type I. Human molecular genetics. 11: 347–357. 10.1093/hmg/11.4.347 11854167

[13] Zinnanti WJ, Lazovic J, Wolpert EB, Antonetti DA, Smith MB, et al (2006) A diet-induced mouse model for glutaric aciduria type I. Brain: a journal of neurology. 129: 899–910. 10.1093/brain/awl009 16446282

[14] Braissant O, Cagnon L, Monnet-Tschudi F, Speer O, Wallimann T, et al (2008) Ammonium alters creatine transport and synthesis in a 3D culture of developing brain cells, resulting in secondary cerebral creatine deficiency. European Journal of Neuroscience 27: 1673–1685. 18380667 10.1111/j.1460-9568.2008.06126.x

[15] Cagnon L, Braissant O (2009) CNTF protects oligodendrocytes from ammonia toxicity: intracellular signaling pathways involved. Neurobiology of Disease 33: 133–142. 18992343 10.1016/j.nbd.2008.09.025

[16] Braissant O, Henry H, Villard AM, Zurich MG, Loup M, et al (2002) Ammonium-induced impairment of axonal growth is prevented through glial creatine. Journal of Neurosciences 22: 9810–9820. 10.1523/JNEUROSCI.22-22-09810.2002 PMC6757846 12427837

[17] Honegger P, Braissant O, Henry H, Boulat O, Bachmann C, et al (2002) Alteration of amino acid metabolism in neuronal aggregate cultures exposed to hypoglycaemic conditions. Journal of Neurochemistry 81: 1141–1151. 12068063 10.1046/j.1471-4159.2002.00888.x

[18] Cagnon L, Braissant O (2008) Role of caspases, calpain and cdk5 in ammonia-induced cell death in developing brain cells. Neurobiology of Disease 32: 281–292. 18722528 10.1016/j.nbd.2008.07.012

[19] Olivera-Bravo S, Fernandez A, Sarlabos MN, Rosillo JC, Casanova G, et al (2011) Neonatal astrocyte damage is sufficient to trigger progressive striatal degeneration in a rat model of glutaric acidemia-I. PloS one 6: e20831. 21698251 10.1371/journal.pone.0020831 PMC3115973

[20] Cagnon L, Braissant O (2007) Hyperammonemia-induced toxicity for the developing central nervous system. Brain Research Reviews 56: 183–197. 17881060 10.1016/j.brainresrev.2007.06.026

[21] Lamp J, Keyser B, Koeller DM, Ullrich K, Braulke T, et al (2011) Glutaric aciduria type 1 metabolites impair the succinate transport from astrocytic to neuronal cells. The Journal of biological chemistry 286: 17777–17784. 21454630 10.1074/jbc.M111.232744 PMC3093853

[22] Amaral AU, Cecatto C, Seminotti B, Zanatta A, Fernandes CG, et al.. (2012) Marked reduction of Na(+), K(+)-ATPase and creatine kinase activities induced by acute lysine administration in glutaryl-CoA dehydrogenase deficient mice. Molecular genetics and metabolism. 10.1016/j.ymgme.2012.04.015 22578804

[23] Ferreira da Costa G, Viegas CM, Schuck PF, Tonin A, Ribeiro CA, et al (2005) Glutaric acid administration impairs energy metabolism in midbrain and skeletal muscle of young rats. Neurochemical research 30: 1123–1131. 16292505 10.1007/s11064-005-7711-9

[24] Ullrich K, Flott-Rahmel B, Schluff P, Musshoff U, Das A, et al (1999) Glutaric aciduria type I: pathomechanisms of neurodegeneration. Journal of inherited metabolic disease 22: 392–403. 10407776 10.1023/a:1005595921323

[25] Quintana A, Kruse SE, Kapur RP, Sanz E, Palmiter RD (2010) Complex I deficiency due to loss of Ndufs4 in the brain results in progressive encephalopathy resembling Leigh syndrome. Proceedings of the National Academy of Sciences of the United States of America 107: 10996–11001. 20534480 10.1073/pnas.1006214107 PMC2890717

[26] Wick R, Scott G, Byard RW (2007) Mechanisms of unexpected death and autopsy findings in Leigh syndrome (subacute necrotising encephalomyelopathy). Journal of forensic and legal medicine 14: 42–45. 16488174 10.1016/j.jcfm.2006.01.002

